# The production, function, and clinical applications of IL-33 in type 2 inflammation-related respiratory diseases

**DOI:** 10.3389/fimmu.2024.1436437

**Published:** 2024-09-05

**Authors:** Shiyao Gu, Ruixuan Wang, Wantian Zhang, Cen Wen, Chunhua Chen, Su Liu, Qian Lei, Peng Zhang, Si Zeng

**Affiliations:** ^1^ Department of Anesthesiology, Sichuan Provincial People’s Hospital, School of Medicine, University of Electronic Science and Technology of China, Chengdu, China; ^2^ School of Medical and Life Sciences, Chengdu University of Traditional Chinese Medicine, Chengdu, China; ^3^ Department of Anatomy and Embryology, School of Basic Medical Sciences, Peking University Health Science Center, Beijing, China; ^4^ Department of Anesthesiology, Affiliated Hospital of Xuzhou Medical University, Xuzhou, China

**Keywords:** IL-33, type 2 inflammation, respiratory diseases, alarmin, asthma

## Abstract

Epithelial-derived IL-33 (Interleukin-33), as a member of alarm signals, is a chemical substance produced under harmful stimuli that can promote innate immunity and activate adaptive immune responses. Type 2 inflammation refers to inflammation primarily mediated by Type 2 helper T cells (Th2), Type 2 innate lymphoid cells (ILC2), and related cytokines. Type 2 inflammation manifests in various forms in the lungs, with diseases such as asthma and chronic obstructive pulmonary disease chronic obstructive pulmonary disease (COPD) closely associated with Type 2 inflammation. Recent research suggests that IL-33 has a promoting effect on Type 2 inflammation in the lungs and can be regarded as an alarm signal for Type 2 inflammation. This article provides an overview of the mechanisms and related targets of IL-33 in the development of lung diseases caused by Type 2 inflammation, and summarizes the associated treatment methods. Analyzing lung diseases from a new perspective through the alarm of Type 2 inflammation helps to gain a deeper understanding of the pathogenesis of these related lung diseases. This, in turn, facilitates a better understanding of the latest treatment methods and potential therapeutic targets for diseases, with the expectation that targeting lL-33 can propose new strategies for disease prevention.

## 1 Introduction

IL-33 is an IL-1 family cytokine produced by various cell types, including epithelial cells, endothelial cells, and fibroblasts (1, 2). Human full-length IL-33 consists of 270 amino acids with a relative molecular weight of approximately 30,000. It contains three functional structural domains: the N-terminal nuclear domain (amino acids 1-65), the central domain (amino acids 66-111), and the C-terminal IL-1-like cytokine structural domain (amino acids 112-270) (3). IL-33 mediates signaling by binding to its receptor ST2 (growth STimulation expressed gene 2), which is predominantly expressed on the surface of various immune cells, such as macrophages, natural killer cells, and certain T cell subsets. IL-33 can participate in T2 immunity by activating ST2-expressing immune cells, including type 2 ILC2, Th2 cells, mast cells, eosinophils, basophils, and dendritic cells (4–11).

Type 2 inflammation is a chronic inflammatory process characterized by persistent activation and dysregulation of the immune system. Unlike acute type 1 inflammation, which typically manifests as rapid and transient inflammation, type 2 inflammation progresses slowly and persists over time. It is commonly observed in various chronic diseases, particularly respiratory conditions like asthma, COPD, pulmonary fibrosis, and allergic rhinitis. Type 2 inflammation involves a complex interplay of inflammation-regulating pathways and cytokines. Key players include Th2 cells, IL-4, IL-13, IL-5, IL-9, thymic stromal lymphopoietin (TSLP), and the IL-33 pathway. These pathways and cytokines play critical roles in orchestrating immune responses in respiratory diseases such as asthma and COPD.

The relationship between type 2 inflammation and IL-33 is significant, as IL-33 is considered a key regulator and “alarmin” in type 2 inflammation, particularly in respiratory diseases. In asthma and other respiratory diseases, IL-33 expression is closely associated with the release of cytokines associated with type 2 inflammation, and overexpression of IL-33 can lead to the development of chronic inflammation and airway remodeling, which can exacerbate the severity of the disease (12), increase respiratory mucus secretion, which can aggravate the symptoms of the disease, and participate in the regulation of the immune homeostasis, which can influence the development and progression of respiratory diseases (13, 14). Thus, IL-33 plays an important role in lung inflammation and is closely associated with type 2 inflammation, influencing disease development and progression by regulating the activation of immune cells and the release of pro-inflammatory factors. This review delves into the pivotal role and function of the IL-33 pathway as an “alarmin” in type 2 inflammation within respiratory diseases, exploring its clinical implications and potential for therapeutic interventions.

## 2 The production of IL-33 in type 2 inflammation in respiratory diseases as “alarmin”

IL-33, also known as high endothelial venous-derived nuclear factor (NF), is a cytokine belonging to the IL-1 family (15). Its primary role involves regulating immune responses, promoting T cell activation, influencing regulatory T cells (Treg), and affecting other immune cells such as macrophages and dendritic cells (16). IL-33 also plays a role in regulating inflammatory responses by promoting the activation of inflammatory cells and enhancing the inflammatory cascade (17). Furthermore, it contributes to tissue repair by promoting wound healing and tissue regeneration (17).

In 2005, Jochen Schmitz and colleagues first proposed that IL-33 could drive Th2responses (18). IL-33 exerts its biological effects through the IL-1 family receptor ST2, which activates NF-κB and MAP kinases, leading to the production of Th2-associated cytokines by polarized Th2 cells *in vitro*. *In vivo*, IL-33 induces the expression of IL-4, IL-5, and IL-13, resulting in severe pathological changes in murine mucosal organs, such as the lung (18). Th2 cells and ILC2s provide protection against worm infections and are involved in allergic reactions; they collaborate at different stages of the immune response (19). Th2 cells primarily induce the expression of IL-25, IL-33, and TSLP through the production of IL-4, while IL-25 and IL-33, in turn, promote the expansion of ILC2s. Therefore, Th2 cell differentiation can occur independently of ILC2s, but the activation of ILC2s can enhance the Th2 response, and Th2 cells can amplify ILC2s by inducing type 2 alarmins (19). The IL-33 signaling pathway in Th2 cells promotes the acquisition of a pro-inflammatory memory state (20). Pathogenic tissue-resident Th2 cells exhibit increased expression of acetyl-CoA carboxylase 1, leading to upregulation of the IL-33 receptor, heightened IL-33 sensitivity, increased IL-5 production, and worsening of airway diseases (21, 22).

The endogenous source of IL-33 is not fully understood; however, it is constitutively active in lymphoid organs, epithelial barrier tissues, brain, and embryos in mice, with epithelial cells being the main source in normal tissues (23). In the human airway, IL-33 is predominantly produced by airway epithelial cells, localized mainly in the nucleus as a full-length precursor. Active full-length IL-33 (IL-33FL) is rapidly released upon cell damage or exposure to environmental stressors like airborne allergens and viruses (24–27).

IL-33FL can be cleaved by serine proteases released by immune cells, such as mast cells, to increase its activity (15). Notably, neutrophil-released elastase, histone G, and protease 3 can also cleave IL-33FL, converting it to a more active, slightly shorter form that is 10-fold more potent (27, 28). Myofibroblasts and endothelial cells may also serve as important sources of IL-33 under inflammatory conditions, while alveolar macrophages can produce high levels of IL-33 in inflamed tissues (23). The release of IL-33 activates Th2 responses, contributing to the onset and progression of inflammation in respiratory diseases.

**Figure 1:**
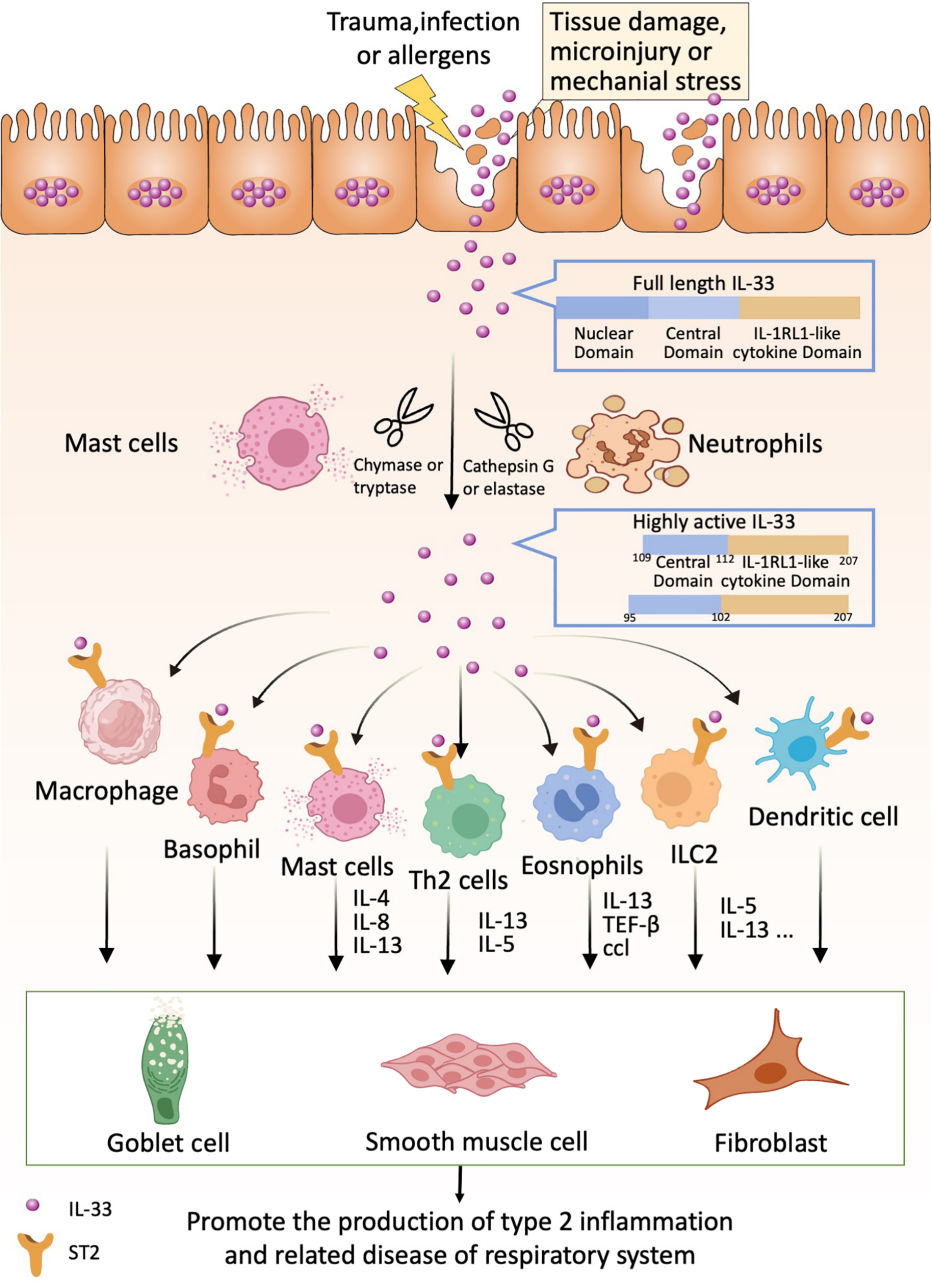
IL-33 acts as an alarmin that can be released following cell or tissue damage. Released full-length IL-33 can be processed into highly active forms by proteases produced by neutrophils and mast cells (e.g., IL-33 95-270 and IL-33 109-270). Highly active forms of IL-33 can bind to ST2 receptors on relevant immune cells such as Th2 cells, basophils, eosinophils, ICL2, dendritic cells, macrophages, etc. These immune cells produce the appropriate cytokines, which in turn act on lung cup cells, smooth muscle cells and fibroblasts to promote lung type 2 inflammation-related diseases.

If we can detect these “alarms” in time and implement appropriate preventive measures proactively, we can effectively reduce and potentially avoid the progression and damage caused by type 2 inflammation in respiratory diseases (**Figure 1**).

## 3 IL-33-related pathways

**Figure 2:**
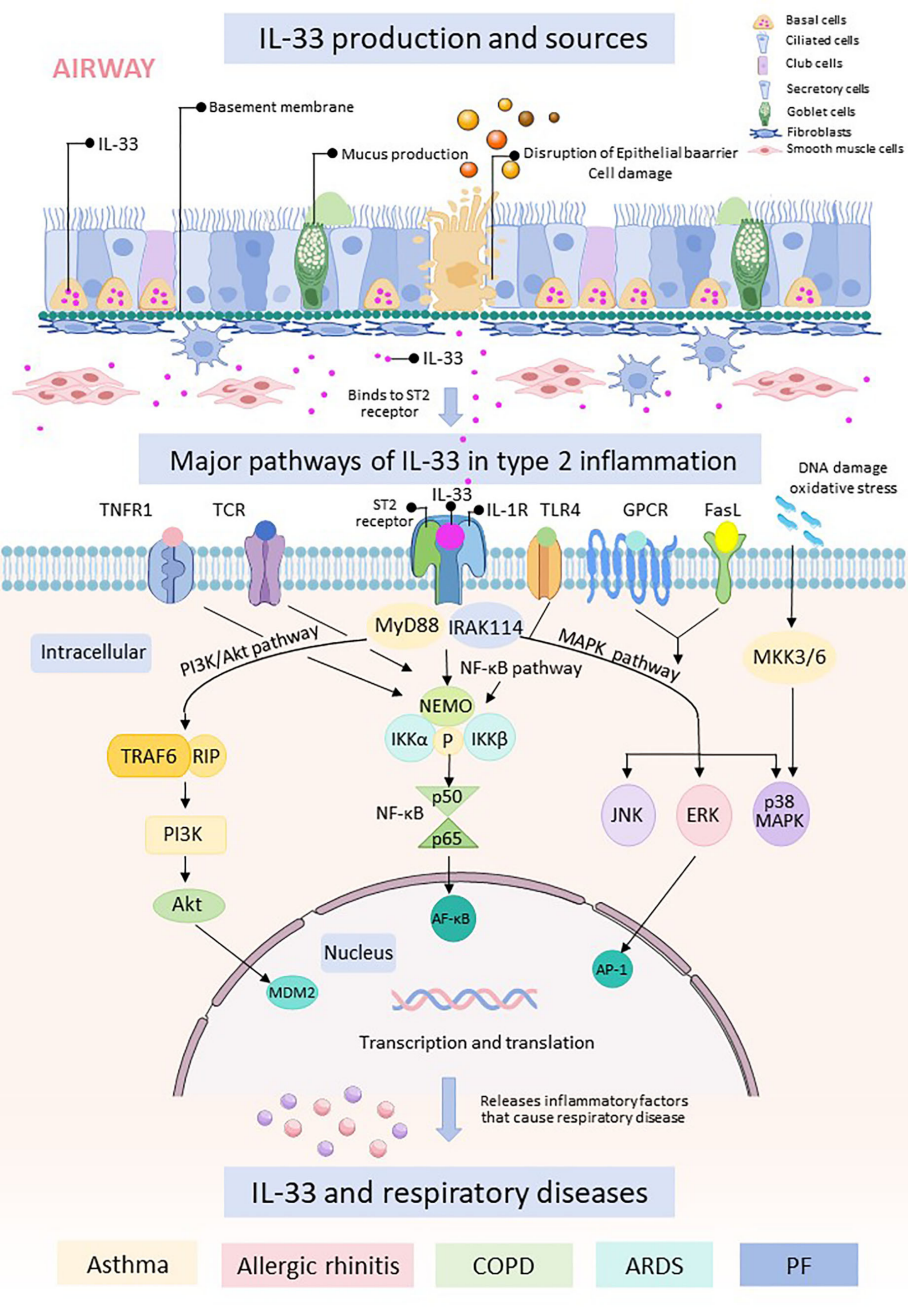
The respiratory epithelial barrier is damaged and releases IL-33. Free IL-33 binds to cells containing ST2 receptors and IL-1R, which can cause intranuclear transcription and translation through three pathways, namely, the PI3K/Akt pathway, the NF-κB pathway, and the MAPK.

IL-33 binds to its receptor ST2 and triggers downstream signaling pathways, including nuclear factor κB (NF-κB), mitogen-activated protein kinases (MAPKs), and phosphatidylinositol 3-kinase/protein kinase B (PI3K/Akt). Activation of these pathways leads to the production of inflammatory mediators, apoptosis, and activation of immune cells, which play critical roles in the development and progression of respiratory diseases. While IL-33 was traditionally thought to be released passively as a result of cell necrosis, emerging evidence suggests additional mechanisms for IL-33 secretion in response to allergens. This secretion may be mediated by purinergic receptor-dependent signaling or through the activation of the dual oxidase 1 Gene-dependent epidermal growth factor receptor (DUOX1-dependent EGFR) signaling pathway (24, 25) (**Figure 2**).

The NF-κB pathway is a central pro-inflammatory signaling pathway involved in regulating the expression of genes encoding cytokines, chemokines, and adhesion molecules. NF-κB refers to a family of transcription factors that play crucial roles in various physiological and pathological processes (29, 30). It responds to oxidative stress and regulates the production and expression of inflammatory cytokines and mediators (31). There are two main pathways of NF-κB activation: the classical and non-classical pathways, each with distinct activation mechanisms (32–35). In the classical NF-κB pathway, the key step involves phosphorylation-dependent activation of the I kappa B Kinases (IKKs) complex (36, 37). Once activated, NF-κB translocates into the nucleus, where it binds to target gene promoters and drives transcription, leading to the expression of inflammatory genes (38, 39). On the other hand, the non-classical NF-κB pathway is activated by specific Tumor Necrosis Factor (TNF) superfamily receptors, suggesting a more specialized biological role for this branch of the pathway (32, 33, 35, 40).

The MAPKpathway consists of three main components: extracellular regulated protein kinases (ERK), c-Jun N-terminal kinase (JNK), and p38 mitogen-activated protein kinase (p38 MAPK) (30). Studies have implicated the p38 MAPK signaling pathway in olfactory loss associated with allergic rhinitis (AR) in animal models (41). Research has shown elevated expression and activity of p38 MAPK mRNA in rats with olfactory loss due to allergic rhinitis compared to control groups (42).

Additionally, the MAPK signaling pathway is involved in regulating IL-33 expression in airway epithelial cells of COPD patients, particularly during viral infections that worsen the condition (12). Physiologically relevant concentrations of IL-33 can activate the MAPK pathway, enhancing cytokine expression in human NK cells, which may influence disease outcomes in conditions like asthma and COPD (43).

In animal experiments, infusion of 1,4NQ-BC into mice triggered lung inflammation and stimulated IL-33 secretion from lung tissues, primarily from macrophages. This process involved both the MAPK and PI3K/Akt signaling pathways, contributing to pro-inflammatory responses in the lungs (44). Furthermore, IL-33 induces and enhances the secretion of IL-6 and IL-8 in human bronchial epithelial cells (HBEs) and peripheral blood mononuclear cells (PBMCs) from COPD patients via the IL-1 family receptor accessory protein (IL-1RAcP) and MAPK pathways, perpetuating chronic inflammation in the respiratory tract (45).

The PI3K/Akt signaling pathway plays a crucial role as an upstream regulator of nuclear factor E2-related factor 2 (Nrf2), which is involved in mediating oxidative stress responses through the Nrf2/HO-1 pathway (46–48). Studies have demonstrated that β-synephrine can modulate oxidative stress by targeting the PI3K/Akt/Nrf2 signaling pathway (49). In allergic rhinitis (AR), elevated serum leptin levels are observed and positively correlate with clinical symptoms (50, 51). Leptin, along with its receptor, activates multiple signaling pathways including MAPK, JAK2-STAT3, and PI3K/Akt pathways (52 , 47). Specifically, in AR, leptin mediates the activity of type 2 innate lymphoid cells (ILC2) and enhances ILC2-driven inflammation through the PI3K/Akt pathway (53).

In epithelial cells from allergic rhinitis (AR) patients, R. Kamekura et al. discovered that treatment with corticosteroids and various inhibitors targeting receptors such as ERK, p38 MAPK, JNK, NF-κB, and epidermal growth factor receptor led to significant inhibition of IL-8 induced by human nasal epithelial cells (HNECs) and IL-33 induced by granulocyte-macrophage colony-stimulating factor (GM-CSF) (54). Moreover, increased intracellular expression of IL-33 may be linked to altered activation of the innate immune receptor Toll-like receptor 4 (TLR4), particularly influenced by cigarette smoke exposure, notably among smokers (55).

In summary, IL-33 plays a pivotal role in type 2 inflammation by engaging multiple signaling pathways and promoting the development of airway inflammation. Targeting these pathways to inhibit IL-33 expression and production has shown promising results in certain clinical trials, offering a new perspective on effective strategies for treating respiratory diseases driven by IL-33 in the future. By understanding the intricate mechanisms through which IL-33 contributes to inflammation, researchers have identified potential therapeutic targets within the NF-κB, MAPK, PI3K/Akt, and other signaling pathways associated with IL-33 activity. Strategies aimed at blocking IL-33 or modulating its downstream effects hold significant therapeutic potential for managing and potentially preventing the progression of respiratory diseases characterized by type 2 inflammation, such as asthma, COPD, and allergic rhinitis. Further exploration and refinement of these targeted approaches are essential to optimize their clinical efficacy and safety, ultimately providing new avenues for personalized treatment and management of IL-33-mediated respiratory conditions.

## 4 IL-33 and type 2 inflammation in respiratory diseases

IL-33 plays a key role in type 2 inflammation, especially in respiratory diseases, where it is implicated in common respiratory diseases such as asthma, allergic rhinitis, chronic obstructive pulmonary disease and acute respiratory distress syndrome.

### 4.1 IL-33 and asthma

Type 2 inflammation is a common feature in patients with asthma, characterized by the activation of group ILC2s in the lungs in response to inhaled allergens, particularly through the production of alarmins such as IL-33 by epithelial cells (56). Activated ILC2s proliferate and secrete IL-5 and IL-13, which contribute to eosinophilic inflammation. In allergic asthma, increased expression of alarmins like TSLP, IL-33, and IL-25 in the airway epithelium is associated with airway obstruction (56). Koji Iijima et al. discovered that IL-33 levels in the lungs of mice rapidly increased within hours after intranasal allergen exposure and continued to rise throughout the chronic inflammation phase. Mice deficient in IL-33R (Il1rl1(-/-)) and thymic stromal lymphopoietin receptor (Tslpr (-/-)) exhibited significantly lower airway inflammation, reduced IgE antibody levels, and decreased airway hyperreactivity (57). Additionally, E. L. Anderson and colleagues found that when naive animals were exposed to airborne allergens, IL-33 in the lungs played a crucial role in promoting the acute bone marrow production of eosinophils. This was achieved through the innate activation of ILC2s and subsequent IL-5 production (58).

Due to the chronic nature of human asthma and challenges in modeling it accurately, researchers primarily rely on mouse models to investigate the role of IL-33 in activating ILC2s and driving type 2 inflammation in the lungs and airways. IL-33 has been shown to activate basophils and mast cells, promoting type 2 inflammation in chronic stable asthma (59). Furthermore, activated mast cells in allergic asthma release serine proteases (chymotrypsin and trypsin) that generate mature, active forms of IL-33, which can potentiate ILC2 activity (15).

IL-33 also stimulates lung CD8 (+) cytotoxic T (Tc) cells to produce type 2 cytokines, which are particularly implicated in severe asthma and asthma exacerbations (60). Increased reprogramming of FoxP3+ regulatory T cells (Tregs) in allergic asthma is associated with IL-33 production, exacerbating airway reactivity (61). Additionally, the RNA-binding protein RBM3 is highly expressed in lung ILCs of asthmatics, induced further by IL-33, and plays a role in regulating ILC function and inflammatory progression (62).

Research has shown that prophylactic or therapeutic administration of P2Y13-R, also known as IL-33 and High-mobility group box1 (HMGB1)’s novel gatekeeper, in experimental asthma models can attenuate the onset and progression of asthma (63). Conversely, intranasal injection of IL-33 or specific allergens in mice leads to increased numbers of ILC2s in the lungs, peripheral blood, and liver. Notably, IL-33-treated mice exhibit proliferation of lung-resident ILC2s and migration of activated ILC2s to the liver, promoting type 2 inflammation and eosinophilic hepatitis (64).

The deterioration of asthma often occurs alongside respiratory viral infections, such as dsRNA challenge or rhinovirus infections, which can exacerbate allergic asthma in mouse models. In models of house dust mite (HDM)-induced asthma, dsRNA attacks can worsen asthma symptoms, with key upstream Th2 cytokines implicated in aggravation being IL-33, TSLP, and IL-25 (65). IL-33 not only induces antiviral signaling in mast cells (MCs) but also upregulates receptors for human rhinoviruses (HRVs), potentially enhancing viral infection and contributing to viral-induced asthma exacerbation (66).

Studies by Kim A.T. Verheijden and colleagues in HDM-induced asthma mouse models demonstrated that adding galacto-oligosaccharides (GOS) to infant formula diets had intestinal and immune modulatory effects, attenuating IL-33 expression associated with intestinal barrier dysfunction (67). Jackson et al. observed elevated IL-33 levels in asthmatics during experimental rhinovirus (RV) progression, correlating with increased levels of T2 cytokines IL-5 and IL-13 in airway mucosal fluid and the severity of worsening following viral inoculation (68). Similarly, Beale and colleagues (69) reported induction of IL-25 by experimental rhinovirus infection, with higher expression levels at baseline and during infection in asthmatic individuals.

In recent years, the morbidity and severity of asthma have increased alongside rising rates of obesity. Mast cells are implicated as targets and sources of various inflammatory factors and stress molecules in response to metabolic burdens, including adipocytokines, IL-9, IL-33, corticotropin-releasing hormone (CRH) and neurotensin (NT) (70). IL-33, in particular, enhances the release of vascular endothelial growth factor triggered by substance P and promotes the release of TNF promoted by neurotensin. IL-9 and IL-33 contribute to pulmonary mast cell infiltration and worsen allergic inflammation, diminishing the response to glucocorticoids and bronchodilators in obese patients (70). In summary, cytokines such as IL-33 play a critical role in the inflammatory response observed in asthmatic patients, particularly contributing to the enhanced type 2 (T2) inflammatory response seen during the aggravation state of asthma. Asthma is a significant health concern, and innovative studies targeting IL-33 could potentially lead to effective treatments for asthma patients.

### 4.2 IL-33 and allergic rhinitis

Interleukin (IL)-33 is a novel member of the IL-1 family of cytokines and acts as a ligand for the IL-1 family receptor, ST2. IL-33 is known to induce a type 2 inflammatory response, which is implicated in allergic inflammation observed in conditions like asthma and atopic dermatitis. In allergic rhinitis, IgE is typically associated with early nasal symptoms such as sneezing and a runny nose, whereas Th2 cytokines are linked to reactions like nasal congestion, discomfort, and irritability (71). However, the specific role of IL-33 and its receptor ST2 in allergic rhinitis remains unclear (54).

Dachuan Fan et al. (72) conducted a study using flow cytometry to quantify the frequency of ILC2s in peripheral blood samples from healthy controls and patients with HDMor mugwort monosensitization. Their findings revealed distinct phenotypic and functional differences in ILC2 frequency among patients with allergic rhinitis sensitized to HDM or mugwort allergens (72). R. Kamekura et al. (54) investigated IL-33 and ST2 expression in normal and allergic rhinitis nasal mucosa using reverse transcription and real-time polymerase chain reaction (PCR), as well as immunohistochemistry. Their results indicated an IL-33-mediated inflammatory response and suggested differential regulation of ST2 by various signaling pathways in human nasal epithelial cells (HNECs) (54). In a randomized, double-blind, placebo-controlled trial involving birch pollen-allergic participants, treatment with birch pollen extract led to significantly greater clinical responses compared to placebo. Notably, participants exhibited a significant increase in levels of soluble IL-33 receptor (sST2) in nasal secretions (73). Additionally, baseline IL-33 mRNA levels were strongly associated with late allergic reactions (LAR) in allergic rhinitis, as revealed in studies investigating the molecular basis of LAR (74). In addition to this, genetic factors including cytokine genes influence IL-33 levels and also play an important role in allergic rhinitis (75, 76). Li et al.’s study provided the first evidence that gene expression profiles of AR-derived nasal fibroblasts (NFs) are associated with IL-33 and IL-6 levels, and BARX1 may be an effective target to alleviate the pathogenesis of AR (77).

On the other hand, no significant differences in IL-25 and IL-33 levels were observed between patients with fungal and non-fungal allergic rhinitis. Chronic fungal exposure may regulate innate systemic cytokines in severe persistent allergic rhinitis (78). Furthermore, in studies using Keyhole Limpet Hemocyanin (KLH) as a neoantigen to induce a Th2 response in humans, no differences in IL-1β and IL-33 expression were observed between control and experimental groups (79). IL-33 is closely associated with allergic rhinitis, and interfering with IL-33 may be a new modality for the treatment of allergic rhinitis.

### 4.3 IL-33 and chronic obstructive pulmonary disease

Senescence is characterized by the gradual decline in lung function due to increased cellular senescence, reduced regenerative capacity, and impaired innate host defenses. The secretion of alarmins, such as IL-33, and the activation of type 2 inflammation represent important mechanisms of innate airway epithelial defense against non-microbial triggers (80). Studies in COPD have demonstrated that exposure to cigarette smoke (CS) promotes IL-33 cytokine responses and contributes to disease development (81). In animal models, CS exposure combined with vascular endothelial growth factor knockout has been shown to recapitulate severe COPD features, including a substantial influx of IL-33-expressing macrophages and neutrophils, highlighting a shift in the CS-induced response towards an uncontrolled, prolonged IL-33-mediated inflammatory reaction from localized surveillance (81). Furthermore, CS-induced pathogenic changes can be notably suppressed by intranasal delivery of neutralizing anti-IL-33 antibodies (82). In summary, IL-33 may play a significant role in influencing the progression of COPD and could be considered a key factor in the disease’s development.

### 4.4 IL-33 and pulmonary fibrosis

PF is a prevalent interstitial lung disease. Recent studies have suggested that IL-33 plays a role in the progression of pulmonary fibrosis, contributing to local inflammation and the formation of fibrotic tissue (83–86). Gao et al. (83) discovered that IL-33 can exacerbate pulmonary fibrosis in mice through the NF-κB pathway. Their experiments inducing pulmonary fibrosis in mice with bleomycin revealed a significant increase in the phosphorylation level of NF-κB p65 protein in lung tissues, along with a marked enhancement in the nuclear translocation of NF-κB p65. Notably, the use of NF-κB inhibitors substantially mitigated these effects (83). Additionally, Yi et al. (87) illustrated that ubiquitin-specific protease 38 (USP38) acts as a negative regulator of IL-33. Their findings demonstrated that mice lacking USP38 displayed more severe pulmonary fibrosis and IL-33-associated lung inflammation following exposure to bleomycin, further solidifying the intricate connection between IL-33 and pulmonary fibrosis (87). Previous research has also indicated that IL-33 can activate transforming growth factor-beta (TGF-β) (86). Recent studies have further suggested that TGF-β1 can induce and sustain the expression of neuropilin-1 (Nrp1), a signaling pathway that upregulates the expression of the IL-33 receptor ST2. This process enhances type 2 immunity and the function of ILC2s, ultimately driving the progression of PF (85). Interestingly, full-length IL-33 seems to have a distinct impact on the pathogenesis of PF compared to processed IL-33. Reports suggest that IL-33 may promote fibrosis development in two forms: full-length IL-33 through both transcription (ST2-dependent) and non-transcription (non-ST2-dependent) mechanisms (88). In conclusion, the relationship between IL-33 and pulmonary fibrosis is intricate, highlighting it as a promising area for further research into the mechanisms and treatment strategies for PF in the future.

Idiopathic pulmonary fibrosis (IPF) is a progressive and highly lethal inflammatory interstitial lung disease characterized by abnormal deposition of the extracellular matrix (89). In the context of idiopathic pulmonary fibrosis, IL-33 may also play a role in its pathogenesis (90). Fanny, M et al. et al. (88) utilized IL-33 in a bleomycin-induced inflammation and Idiopathic pulmonary fibrosis model using mouse IL-33 receptor [tumorigenic 2 (ST2) chain-inhibited] mice compared to C57BL/6 wild-type mice. Unexpectedly, it was found that acute neutrophilic lung inflammation led to the development of IL-33/ST2-dependent pulmonary fibrosis associated with M2-like polarization production, and that 24 hours after bleomycin treatment, ST2-deficient mice exhibited enhanced inflammatory cell recruitment, and MRI showed enhanced inflammation in the lungs with pulmonary edema (91). Lee et al. (90) demonstrated that levels of IL-33 and TSLP in bronchoalveolar lavage fluid may help differentiate IPF from other chronic interstitial lung diseases. Another research has shown that the pro-fibrotic role of IL-25/IL-33/TSLP in IPF represents a novel paradigm, acting directly on two key cells in the fibrotic process: alveolar epithelial cells and (myo)fibroblasts (84).Additionally, Majewski et al. (92) analyzed exhaled breath condensate from IPF patients and found the presence of IL-33, suggesting a potential association between IL-33 and the progression of IPF. Interestingly, full-length IL-33 can contribute to the pathogenesis of IPF by promoting the expression of cytokines such as TGF-β, interleukin-6 (IL-6), monocyte chemotactic protein-1 (MCP-1), macrophage inflammatory protein-1α (MIP-1α), and tumor necrosis factor-alpha (TNF-α) (93). This, in conjunction with bleomycin, leads to the accumulation of lung lymphocytes and collagen production in IPF (93). In contrast, Katherine E. Stephenson et al. utilized a mouse model of bleomycin (BLM)-induced pulmonary fibrosis and evaluated the fibrotic potential of the IL-33-mediated ST2 signaling pathway using a therapeutic dose of ST2-Fc fusion protein. Their findings suggested that this pathway is unlikely to exert a central fibrotic effect in idiopathic pulmonary fibrosis (94). However, further research is needed to elucidate the specific pathways involved. Future studies can build upon this foundation to delve deeper from a broader perspective, increasing the diversity and reliability of research data to contribute to the understanding of the mechanisms and treatment of IPF.

Moreover, IL-33 has been implicated in pulmonary fibrosis associated with systemic sclerosis (SSc). Previous studies have suggested that endothelial cell damage in early SSc patients upregulates IL-33 mRNA expression, leading to the release of IL-33 protein into the bloodstream (95, 96). Recent research has demonstrated elevated levels of IL-33 in SSc patients, with correlations observed between IL-33 levels and the severity of skin sclerosis and pulmonary fibrosis (97, 98). Furthermore, varying degrees of IL-33 upregulation have been identified in patients with cystic fibrosis and silicosis, indicating a potential role for IL-33 in the pathogenesis of these diseases, necessitating further investigation in the future (99, 100).

The treatment of pulmonary fibrosis, encompassing a wide range of diseases, has long posed a significant challenge for the medical community. While numerous research findings have laid the groundwork for understanding these conditions, there remain numerous unexplored avenues that warrant thorough investigation. Advancing our understanding through comprehensive exploration and the accumulation of reliable evidence will be pivotal in unraveling the mechanisms underlying pulmonary fibrosis and related disorders, ultimately aiding in the development of effective treatments in the future.

### 4.5 IL-33 and other respiratory diseases

In studies of acute respiratory distress syndrome (ARDS), IL-33 has been shown to play a critical role in modulating post-injury inflammation by controlling local cytokine levels and populations of Foxp3+ Tregs (101). Additionally, research in a mouse model of LPS-induced lung injury has revealed that early release of IL-33 in ARDS contributes to an uncontrolled inflammatory response through activation of Invariant natural killer T cells (iNKT cells) (102). This suggests that IL-33 and NKT cells could potentially serve as targets for early intervention in cytokine storms associated with ARDS (102). Brandon W. Lewis et al. studying a cystic fibrosis-like lung disease model, compared IL-33 gene knockout (IL-33KO) Tg+ mice with IL-33 heterozygous (IL-33HET) Tg+ mice. They found that IL-33KO/Tg+ mice had completely absent eosinophils in bronchoalveolar lavage fluid compared to IL-33HET/Tg+ mice, and mucin staining within airway epithelial cells was completely lost. Additionally, levels of IL-5, IL-4, and Th2-related gene markers (Slc26a4, Clca1, Retnla, and Chi3l4) were significantly reduced, indicating that mucus obstruction was not dependent on IL-33 (103). Moreover, investigations have explored TNFα-induced cytokine production in primary lung fibroblasts from individuals with asthma compared to non-asthmatics, demonstrating significant upregulation of IL-6, IL-8, C-C motif chemokine ligand 5 (CCL5), and TSLP mRNA expression and protein secretion in lung fibroblasts upon TNFα stimulation (104). While numerous studies have confirmed the association of IL-33 with respiratory diseases such as asthma, allergic rhinitis, COPD, and pulmonary fibrosis limited research has investigated the role of IL-33 in other respiratory conditions like ARDS. Consequently, there remains ambiguity regarding the involvement of IL-33 in these diseases, highlighting the need for further studies to elucidate its role and therapeutic implications. Additional investigations are warranted to clarify the relationship between IL-33 and various respiratory disorders beyond the well-established conditions, paving the way for more targeted therapeutic approaches and a comprehensive understanding of IL-33 biology in respiratory pathology.

## 5 Current status of treatment of type 2 inflammatory respiratory diseases

The treatment of respiratory diseases is confronted with several challenges, encompassing complex etiologies, drug resistance concerns, inflammation, and airway remodeling, as well as individual patient variations such as immune system variability. Moreover, challenges also include issues related to long-term therapy adherence, and the cost and accessibility of treatment. At the heart of type 2 inflammation is the production of cytokines and other inflammatory mediators by T-lymphocytes. These molecules play a pivotal role in the initiation and perpetuation of respiratory diseases by inciting inflammatory responses and influencing the disease trajectory. Addressing these challenges requires a multifaceted approach that integrates insights from immunology, pharmacology, and personalized medicine. Novel therapeutic strategies that specifically target key mediators of inflammation, including cytokines produced by T-lymphocytes, offer promising avenues for improving treatment outcomes and addressing the complexities inherent in managing respiratory diseases. Additionally, efforts to enhance treatment adherence and optimize therapy accessibility are essential for achieving effective disease management and improving patient quality of life.

### 5.1 Asthma

Asthma is a complex and challenging disease characterized by diverse etiologies and mechanisms. It encompasses various clinical phenotypes, including allergic asthma, nonallergic asthma, adult-onset asthma, asthma with persistent airflow limitation, and obesity-related asthma. Additionally, asthma can be classified based on sputum granulocyte infiltration proportions, such as eosinophilic, neutrophilic, mixed granulocyte, and oligophilic types. The prevalence of asthma is significantly influenced by age and gender. In children, males under thirteen years old tend to have a higher incidence compared to females, whereas in adulthood, females exhibit a higher incidence than males (105–107). Currently, the most effective long-term treatment for asthma management is inhaled corticosteroids (ICS) (108). However, despite high-dose ICS therapy and combination with long-acting β2 agonists or leukotriene modulators, some patients struggle to control their condition. This challenge may be attributed to the heterogeneous nature of asthma, where patients with different asthma phenotypes do not uniformly respond to standard anti-asthma treatments (109). Therefore, further studies are still needed to explore effective drugs and provide new ideas for the treatment of asthma.

#### 5.1.1 Blocking IL-33 production and release in asthma therapy

Fruit and vegetable intake has been associated with a reduced risk of asthma and improved lung function, particularly evidenced by enhanced Forced Expiratory Volume in the first second (FEV1) and decreased wheezing frequency, with notable benefits observed from consuming apples and oranges (110–112). These foods are rich sources of essential vitamins, including vitamin A, vitamin C, vitamin D, and vitamin B6, each of which contributes to protective effects against asthma development (113–116). Recent studies highlight the effectiveness of vitamin B6, particularly its active form pyridoxal phosphate (PLP), in alleviating type 2 inflammatory responses and reducing asthma severity. Healthy individuals tend to exhibit higher concentrations of PLP compared to asthmatics, with the lowest PLP levels observed in patients with severe asthma. Interestingly, serum PLP concentrations positively correlate with FEV1 and negatively correlate with eosinophil levels in asthmatics, indicating a protective role of vitamin B6 in asthma progression (116). Moreover, research by Zhu et al. revealed that PLP inhibits MDM2-mediated ubiquitination of IL-33, a crucial step for IL-33 stability and type 2 inflammatory response regulation. By promoting IL-33 degradation through ubiquitin modification, vitamin B6 attenuates inflammatory responses associated with asthma (116). Given the accessibility and affordability of vitamin B6 through dietary sources, it represents a promising and cost-effective therapeutic strategy for asthma treatment. Incorporating vitamin B6-rich foods into daily nutrition may offer a safe and accessible approach to mitigating asthma symptoms and improving overall respiratory health. Continued research in this area holds potential for advancing personalized nutritional interventions in asthma management.

Upstream of IL-33, drugs primarily act by inhibiting IL-33 expression and activation. Increased SPRR3 gene expression in asthma patients significantly elevates IL-33 levels. Inhibiting SPRR3 suppresses IL-33 expression in lung tissues and bronchoalveolar lavage fluid (BALF) of asthmatic mice. This inhibition further dampens the activation of the PI3K/Akt/NFκB pathway, reduces expression of IL-25 and TSLP, decreases recruitment of ILC2 cells, and weakens Th2 immune responses (117). Additionally, ascr#7, found in ascarosides produced by parasites like N. brasiliensis, in combination with antigen/gelatin, reduces IL-33 expression in lung epithelial cells. By preventing downstream effector activation by IL-33, it inhibits IL-33-mediated proliferation of ST2+Th2 cells and ILC2 cells (118). The miR-206/cd39/ATP axis regulates airway IL-33, IL-25, and TSLP expression and type 2 inflammation. Elevated ATP levels activate extracellular purinergic receptors, which mediate the release of IL-33. Reduced ATP levels inhibit IL-33, IL-25, and TSLP release, as well as downstream ILC2 amplification (119). Wowbaine, an inhibitor of sodium-potassium ATPase, suppresses the production of IL-33, TSLP, IL-1β, and IL-4 in OVA mice. It also decreases levels of tissue remodeling markers like TNF-α and TGF-β, inhibits cell migration, and reduces vascular permeability (120). Jina et al. (121) found that intranasal inhalation of IFN-λs accelerates the reduction of IL-33 and TSLP levels in the lungs of asthmatic mice, alleviating lung injury induced by Th2 responses. This approach represents a potential method to control Th2-mediated allergic reactions.

Chinese medicines have a long history of use in treating asthma, and recent studies suggest that some Chinese medicines may target the IL-33 pathway upstream. Louki Zupa (LKZP) has been found to inhibit the expression of IL-33 and ST2 in OVA mice. It suppresses the IL-33/ST2-NFκB/GSK3β/mTOR pathway-mediated inflammatory response, thereby attenuating airway inflammation in OVA mice (122). Irisflavin A, another compound, exhibits chemoprotective effects. Treatment of OVA mice with irisflavin A reduces IL-33, IL-4, and IL-5 levels along with their mRNA expression. This treatment also decreases airway hyperresponsiveness in asthmatic mice and exerts a protective effect against airway inflammation and eosinophil chemotaxis (123). Furthermore, in mice sensitized with HDM and undergoing dietary intervention with galacto-oligosaccharides (GOS), levels of IL-33 and ST2 were reduced, and the distribution of IL-33 was altered, leading to a weakened inflammatory response (67).

Another study identified a positive correlation between plasma thrombin-antithrombin complex (TATc) levels and the number and function of innate lymphoid cell type 2 (ILC2) in PBMCs of allergic patients (124). Direct or indirect thrombin inhibitors, such as bivalirudin (TFA) and low molecular weight heparin, were found to inhibit the IL-33-associated type 2 immune response by affecting IL-33 maturation and ILC2 activation *in vivo*. These findings suggest a potential therapeutic approach involving thrombin inhibition to modulate IL-33-mediated immune responses in allergic conditions.

#### 5.1.2 Blocking IL-33/ST2 binding and downstream conduction pathways in asthma therapy

Downstream of the IL-33 pathway, drugs can block IL-33 binding to ST2 and inhibit ILC2 production, thereby attenuating the type 2 inflammatory response. The interleukin family is involved in IL-33-induced allergic asthma, with IL-9 being a downstream cytokine of IL-33. Studies in IL-9-deficient mice have shown that IL-33-induced airway hyperresponsiveness, inflammatory cell infiltration, cuprocyte chemotaxis, collagen deposition, smooth muscle hypertrophy, and downstream cytokine expression were all attenuated, suggesting the involvement of IL-9 in IL-33-induced airway inflammation and asthmatic airway remodeling (125). Mast cells are known to produce interleukin 9 (IL-9), IL-33, and stress molecules. IL-33, in turn, induces mast cells to produce IL-13, which promotes mast cell survival and enhances allergic inflammation by promoting lung mast cell infiltration. Certain flavonoids, such as quercetin and lignans, exhibit potent mast cell inhibitory effects along with anti-inflammatory and antioxidant properties, suggesting a promising therapeutic approach (126, 127). Additionally, blocking the IL-33/RAGE/VCAM-1 pathway inhibits IL-33-induced ILC2-mediated type 2 inflammatory response, leading to the elimination of eosinophilic inflammation and mucous epithelial hyperplasia (128). The mTOR (rapamycin signaling) pathway also plays a role in asthma, where IL-33 induces phosphorylation of rpS6 in bone marrow ILC2s via mTORC1 signaling to initiate the immune response. Rapamycin may attenuate IL-33-induced eosinophilic inflammation by inhibiting IL-5-induced bone marrow ILC2s and reducing mTOR signaling (129). Furthermore, activation of ILC2s downstream of IL-33 and allergic airway inflammation can be inhibited through the adenosine/A2A signaling pathway, likely due to adenosine binding to its receptor, which increases intracellular cAMP production and downregulates the downstream NF-κB pathway (130).

In obese asthma patients, treatment with glucagon-like peptide-1 receptor agonist (GLP-1RA) led to reduced acute release of IL-33 and TSLP, decreased airway hyperresponsiveness, and attenuation of ILC2 activation, providing a potential therapeutic direction for obese asthma treatment (131). Cyclohexane di-gmp (CDG) (132) modulates the downstream ILC2 response to ALT-induced IL-33 release, effectively reducing ALT-induced eosinophilia in bronchoalveolar lavage (BAL) and lungs, along with decreasing IL-5 and IL-13 levels in BAL, attenuating ILC2-driven eosinophilic airway inflammation. Another traditional Chinese medicine, serpentine (133), has been shown to reduce airway hyperresponsiveness and attenuate airway inflammatory response and remodeling by inhibiting IL-33/ST2 signaling, balancing Th1/Th2-associated cytokines, and achieving therapeutic efficacy in mice at 50 mg/kg. Moreover, Fibulin-1 (Fbln1), an ECM protein, stabilizes the deposition of other ECM proteins and collagen. Deletion of Fbln1c in a mouse model of chronic experimental asthma demonstrated protective effects against elevated IL-33, IL-5, IL-13, and TNF, reduced airway remodeling and hyperresponsiveness (AHR), decreased recruitment of neutrophils, eosinophils, and lymphocytes, and lower levels of airway inflammation-related cytokines and chemokines, suggesting a potential therapeutic target for inhibiting chronic asthma airway remodeling and inflammation. Lastly, in patients with mild allergic asthma who discontinued glucocorticoid therapy, the ratios of IL-1β to IL-37 and IL-33 to IL-37 were significantly elevated compared to asthmatics, potentially underlying the persistence of allergic asthma in adults. IL-37 suppresses allergic airway inflammation by balancing the disease-amplifying effects of IL-1β and IL-33 (134). However, the specific mechanism remains unclear, necessitating further studies to elucidate causation and therapeutic potential.

#### 5.1.3 Monoclonal antibodies in asthma therapy

Blocking TSLP, ST2, and IL-33 can all be effective in treating asthma. Tezepelumab, a monoclonal antibody targeting TSLP, works by directly binding to TSLP and blocking TSLP-TSLPR signaling. It has shown efficacy and long-term safety in treating severe asthma (135). Astegolimab selectively inhibits ST2, reducing the rate of acute asthma deterioration and demonstrating safety and efficacy in patients with severe T2-low type asthma. Antibodies targeting IL-33 that have been studied include itepekimab, tozorakimab, and REGN3500. Although many of these drugs are still in the clinical evaluation stage, itepekimab has demonstrated a significant reduction in the odds of uncontrolled asthma compared to placebo (136). Tozorakimab acts by blocking both IL-33(red) and IL-33(ox) signaling, providing a new strategy for treating inflammation and epithelial dysfunction (137). Interestingly, Vannella et al. (138) studied IL-33, IL-25, and TSLP using the Schistosoma haematobium egg lung granuloma model of allergic airway disease and concluded that targeting only one cytokine is useful but incomplete for reducing allergic airway disease. They suggested that targeting all three cytokines simultaneously could be more meaningful (138). Further studies are needed to confirm the roles of these drugs and provide safer options for pharmacologic asthma treatment.

#### 5.1.4 Allergen immunotherapy in the treatment of asthma

Allergen Immunotherapy (AIT) stands as a pivotal element within asthma treatment, attracting considerable attention within the medical community in recent years, with well-defined international guidelines in place (139). In a retrospective study conducted by Wang et al. involving 112 children diagnosed with allergic rhinitis (AR) and cough variant asthma (CVA), Subcutaneous Immunotherapy (SCIT) was shown to forestall the emergence of new sensitizations and the progression to classic asthma (CA) in this specific pediatric cohort. Moreover, SCIT treatment demonstrated enhancements in serum levels of sIgG4, IL-27, and IL-33, yielding superior clinical outcomes compared to traditional pharmacological approaches (140). Additionally, a study indicated the potential of allergen immunotherapy in reducing the incidence of respiratory infections and mitigating the risk of acute exacerbations in individuals with asthma. Christian and colleagues’ (141) research revealed that House Dust Mite Sublingual Immunotherapy (HDM-SLIT) significantly upregulates the expression of bronchial epithelial IFN-β and IFN-λ, thereby dampening the response of IL-33 to the viral mimic poly(I:C). These findings suggest that this therapeutic approach may bolster tolerance to viral infections and enhance the stability of airway epithelium.

In summary, IL-33 plays a critical role in the early stages of asthma development by initiating downstream signaling pathways that contribute to asthma induction and exacerbation. Numerous drugs have demonstrated the ability to lower IL-33 levels and alleviate asthma symptoms. However, the precise mechanisms and targets involved in IL-33 down-regulation remain unclear, necessitating further research to identify these mechanisms and targets. This deeper understanding will pave the way for novel therapeutic options for the treatment of asthma.

### 5.2 Allergic rhinitis

Given the extensive research on the role of IL-33 in allergic rhinitis (AR) in recent years, targeting IL-33 represents a valuable approach to intervening in the onset and progression of AR (142). In addition to traditional treatments like antihistamines, glucocorticoids, and allergen avoidance, understanding and modulating the IL-33 pathway could offer novel strategies for managing allergic rhinitis effectively. This approach leverages insights into the specific mechanisms underlying AR pathogenesis, potentially leading to more targeted and impactful therapeutic interventions.

#### 5.2.1 Blocking IL-33 production and release in allergic rhinitis

Xiaoqinglongtang (XQLT) is a traditional Chinese herbal formula described in the Shang Han Lun, comprising ephedra, peony, and xixin. Manabu and colleagues (143) found that XQLT administration helped control symptoms and reduced IL-33 levels in serum and nasal lavage fluid in TDI-induced AR mice. Further investigations revealed that XQLT could decrease IL-33 release from nasal epithelial cells. Zhang et al. (144) studied the mechanism of XQLT *in vivo* and discovered that XQLT significantly reduced nasal mucosal tissue damage in an OVA-induced AR mouse model. They observed decreased levels of IL-33, ST2, MYD88, and NF-κB p65 proteins in the XQLT intervention group compared to the control group, suggesting that XQLT might inhibit the IL-33/ST2 signaling pathway through the MYD88/IRAK4/NF-κB p65 pathway (145). Rezwanul et al. (146) demonstrated that wild grape extracts could inhibit IL-33 gene expression in HeLa cells and Swiss 3T3 cells, indicating that wild grapes may contain compounds that inhibit IL-33 expression. Additionally, they found that betuletol, extracted from Brazilian propolis, reduced eosinophil numbers by inhibiting ERK phosphorylation and down-regulating IL-33 expression, thereby improving eosinophilic inflammation symptoms. Some of these substances are already used clinically, while others are still in the laboratory research phase. Further clinical studies are necessary to validate their effectiveness and safety for treating allergic rhinitis and related conditions.

#### 5.2.2 Blocking IL-33/ST2 binding and downstream conduction pathways in allergic rhinitis

MicroRNAs (miRNAs) are small endogenous non-coding molecules that play a crucial role in post-transcriptional regulation (147). Studies have demonstrated that miR-181a-5p can target IL-33, leading to its downregulation and inhibition of the IL-33/ST2/p38 MAPK axis, exerting an anti-inflammatory effect in human RPMI-2650 nasal epithelial cells (148). Another study has shown that Didox reduces cytokine production following IL-33 activation by inhibiting NFκB and AP-1 transcriptional activity in primary mouse mast cells, suggesting that Didox could potentially act as a treatment for ARpatients (142, 148). Additionally, Jin et al. (149) found that Chaenomeles sinensis extract (CSE) could improve rhinitis symptoms in OVA-induced AR mice. Immunohistochemistry revealed that mice gavaged with CSE showed more restricted distribution of ST2 compared to positively treated group mice, along with reduced IL-33 levels, indicating that CSE may be effective in controlling IL-33/ST2 axis-mediated cellular inflammation in nasal epithelial cells. Furthermore, FJE (a yellow flower root extract) is commonly used for allergic inflammation treatment and has been reported to modulate mucus accumulation by reducing inflammatory cell infiltration. It can also inhibit OVA-specific immunoglobulins and modulate the IL-33/TSLP/NF-κB signaling pathway, thereby exerting therapeutic effects on allergic rhinitis (150).

#### 5.2.3 Allergen immunotherapy in the treatment of allergic rhinitis

The pathogenesis of allergic diseases primarily involves allergen-specific IgE and Th2-related cellular inflammation. Allergen-specific immunotherapy primarily targets cellular immunity by inhibiting the production and release of IL-25 and IL-33, reducing the recruitment of inflammatory cells such as ILC2 and eosinophils, and suppressing Th2-related cellular inflammation. This, in turn, alleviates allergic airway inflammation and airway hyperresponsiveness (151, 152). The severity of allergic rhinitis (AR) is positively correlated with IL-33 levels, and a decrease in its levels may indicate improvement in clinical symptoms. Therefore, IL-33 could be considered a crucial predictive factor and a key therapeutic target in the treatment of AR (153, 154).

### 5.3 Chronic obstructive pulmonary disease

COPD is a prevalent respiratory condition, and emerging research indicates the involvement of type 2 cytokines, including IL-33, in its development (155–157). Current clinical approaches for COPD management primarily involve M-cholinergic inhibitors, β-adrenergic receptor agonists, and glucocorticoids (158). However, specific treatments targeting type 2 inflammation in COPD, such as those involving IL-33, remain lacking. Recent studies have highlighted potential therapeutic strategies targeting IL-33 for COPD. These may include interventions aimed at modulating IL-33 expression, blocking IL-33 signaling pathways, or targeting downstream effectors of IL-33-mediated inflammation. Investigating IL-33 as a therapeutic target could open avenues for developing more tailored and effective treatments for COPD associated with type 2 inflammation (159–164).

#### 5.3.1 Blocking IL-33 production and release in chronic obstructive pulmonary disease

Previous studies have shown that the serum amyloid A (SAA)/IL-33 axis plays a role in the pathological process of steroid-resistant pulmonary inflammation (159). Recent research has indicated that bitterside II could be a potent inhibitor of SAA and IL-33 production. Bitter Glycoside II, a metabolite derived from the plant extract mullein, was found to inhibit LPS-induced SAA1 mRNA expression in human monocytes and SAA-induced IL-33 secretion in human airway epithelial cells. Further investigations revealed that this compound inhibited the TLR2-MAPKp38-ERK1/2 and NF-κB signaling pathways activated by SAA, thereby suppressing IL-33 production and potentially controlling airway remodeling in COPD patients (159). These findings suggest that bitterside II holds promise as a therapeutic agent, although its safety and efficacy require validation through additional animal and clinical trials.

Ella and colleagues (160) observed a significant increase in the secretion of the ceramide biosynthetic enzyme neutral sphingomyelinase 2 (nSMase2) and the IL-33 isoform IL-33Δ34 in patients with COPD following a comprehensive analysis of human airway epithelial cells. Subsequent investigations revealed that nSMase2 promotes IL-33 secretion by integrating into the multivesicular endosomal (MVE) pathway, thereby exacerbating COPD severity in humans. They conducted experiments using the nSMase2 inhibitor, GW6849, which effectively suppressed IL-33 secretion and downstream inflammatory pathways, demonstrating its potential as a novel therapeutic approach for COPD treatment. However, as of now, no relevant drugs targeting this pathway have entered clinical trials.

#### 5.3.2 Blocking IL-33/ST2 binding and downstream conduction pathways in chronic obstructive pulmonary disease

IL-33 plays a role in accelerating the progression of COPD by inducing and enhancing the expression of IL-6 and IL-8 inHBE cells and PBMCs of COPD patients via the ST2/IL-1RacP pathway and MAPKs pathway. In a study conducted by Jin et al. (45), the existence of this pathway was confirmed through cytological experiments. They demonstrated that the expression of IL-6 and IL-8 could be down-regulated by administration of ST2-neutralizing antibody, IL-1RACP-neutralizing antibody, the MAPK inhibitor SB-203580, or the JNK inhibitor SP-600125. These findings suggest that inhibitors targeting these pathways may hold potential therapeutic effects for COPD.

#### 5.3.3 Monoclonal antibodies in chronic obstructive pulmonary disease

Itepekimab, a monoclonal antibody targeting IL-33, has shown potential for COPD treatment in a multicenter, randomized, double-blind, phase 2a clinical trial. In this trial, patients already receiving disease-controlling therapy were additionally treated with Itepekimab (300 mg per injection, twice every two weeks for 24-52 weeks). Subgroup analysis of ex-smokers revealed a 42% reduction in the rate of acute COPD worsening and an improvement in FEV1 by 0.09 L (161). These results suggest potential benefits in lung function improvement for ex-smokers and usefulness in COPD treatment. Astegolimab, a monoclonal antibody against ST2, was investigated in a clinical trial conducted by Aj and colleagues (162). While it did not control the acute exacerbation rate in COPD patients, it showed promise in improving respiratory function and quality of life. Notably, improvements in Saint George’s Respiratory Questionnaire (SGRQ-C) and FEV1 were more significant in patients with higher eosinophils compared to those with lower eosinophils. The study also observed significantly lower blood and sputum eosinophil counts in the drug group compared to the placebo group, contributing to disease control. Further experimental and clinical studies are warranted to explore the potential of anti-ST2 therapy in COPD remission. Tozorakimab, a novel monoclonal antibody, acts by blocking IL-33 formation via the ST2 pathway and inhibiting its signal transduction in the RAGE/EGFR pathway (137). In a recent clinical study by F et al. (163), Tozorakimab demonstrated dose-dependent elevation of serum IL-33/Tozorakimab conjugates and decreased IL-33/ST2 conjugates compared to placebo. The experimental group showed significantly lower serum IL-5 and IL-13 levels and reduced blood eosinophil levels in COPD patients across all dose levels. The safety and tolerability of the drug were also confirmed. Although respiratory function was not examined in this study, ongoing phase 2 and phase 3 clinical trials aim to address these aspects and provide further insights into the efficacy of Tozorakimab for COPD treatment.

#### 5.3.4 Other treatment mechanisms in chronic obstructive pulmonary disease

In COPD treatment regimens, glucocorticoids are considered the most widely used anti-inflammatory agents (164); however, in recent years, many patients have been found to exhibit decreased sensitivity to glucocorticoids, and IL-33 is considered to be a mediator of steroid resistance (165). Recent studies have shown that azithromycin and budesonide, when used alone, cannot inhibit the release of IL-33 from peripheral monocytes, but their combination can reduce the release of IL-33. These findings suggest that azithromycin helps to restore sensitivity to steroids in COPD patients, providing an adjunctive option for disease treatment (164).

### 5.4 Pulmonary fibrosis

Pulmonary fibrosis represents the end-stage of a lung condition characterized by fibroblast proliferation and extensive accumulation of extracellular matrix, accompanied by inflammatory damage and structural deterioration (166, 167). IL-33, a novel pro-fibrotic cytokine, signals through ST2, facilitating the onset and progression of pulmonary fibrosis by recruiting and guiding the functions of inflammatory cells in a ST2 and macrophage-dependent manner, and amplifying the production of pro-fibrotic cytokines. Attenuating and treating pulmonary fibrosis can be achieved by weakening or blocking its upstream and downstream pathways (166, 167). Bleomycin (BLM) is a critical chemotherapeutic agent used in cancer treatment. However, its cytotoxicity, associated with DNA strand scission and induction of reactive oxygen species, can lead to severe side effects, including pulmonary fibrosis (168–170). IL-33 plays a significant role in the initiation and progression of pulmonary fibrosis. The levels and activity of the IL-33 receptor (IL-33R) are regulated through deubiquitination by ubiquitin-specific protease 38 (USP38) and polyubiquitination by E3 ubiquitin ligase tumor necrosis factor receptor–associated factor 6 (TRAF6) (87). USP38 mediates the deubiquitination of IL-33R via the autophagy-lysosome pathway, promoting receptor downregulation, negatively regulating IL-33-triggered inflammatory signaling, and the inflammatory response; following bleomycin induction, mice with USP38 deficiency exhibit significantly increased transcription of inflammation and fibrosis-related genes in the lungs, heightened inflammatory cell infiltration, collagen deposition, elevated levels of the fibrosis marker α-SMA, and increased mortality (87). Upon IL-33 stimulation, TRAF6 enhances K27-linked polyubiquitination of IL-33R, preventing its autophagic degradation, promoting IL-33R stability, and facilitating effective signal transduction (87). Therefore, USP38 and TRAF6 represent potential drug targets for addressing IL-33-induced pulmonary fibrosis. A study by Zhang JJ et al. (85) revealed that neuropilin-1 (Nrp1) acts as a tissue-specific regulator for lung-resident ILC2. The transforming growth factor beta-1 (TGFβ1)-Nrp1 signaling pathway enhances ILC2 function and type 2 immunity by upregulating IL-33 receptor ST2 expression. Targeting Nrp1 can inhibit the activation of lung ILC2 and attenuate the pulmonary fibrotic response (85). Additionally, eukaryotic initiation factor 3a (eIF3a) serves as a crucial regulatory factor in fibrotic diseases (171, 172). It has been reported that IL-33 can activate the NF-κB pathway to induce eIF3a expression, and the NF-κB pathway inhibitor pyrrolidine dithiocarbamate (PDTC) can reverse this reaction, thereby inhibiting the development of pulmonary fibrosis (173–177). Another pro-apoptotic agent, Dehydrocostus lactone (DHL) (173–177), can downregulate the JNK/p38 MAPK-mediated NF-κB signaling pathway, inhibit macrophage activation, and exert anti-fibrotic effects (178).

Idiopathic pulmonary fibrosis (IPF) is a progressive, irreversible lung disease characterized by respiratory distress and respiratory failure primarily caused by the formation and thickening of lung interstitial tissue scars (179, 180). Currently, pirfenidone and nintedanib are the only treatment options for IPF (179, 180). Researchers are actively exploring new therapeutic drugs and targets. Elamipretide (SS-31) is a novel insulin-targeted antioxidant that protects and repairs damaged mitochondria without affecting healthy mitochondria (181). SS-31 offers protection against pulmonary fibrosis and inflammation by inhibiting the activation of the NLRP3 inflammasome in macrophages mediated by Nuclear factor erythroid 2-related factor 2 (Nrf2) (89). Nuclear factor of activated T cells cytoplasmic member 3 (NFATc3) responds to damaged epithelial cells, IL-33, and Th2 cell stimuli, promoting the production of C-C motif ligand 2 (CCL2) and C-X-C motif chemokine ligand 2 (CXCL2), exacerbating early inflammation, regulating angiogenesis, mediating fibroblast recruitment, and participating in the pathogenesis of pulmonary fibrosis (89). NFATc3 deficiency significantly reduces bleomycin-induced pulmonary fibrosis and inflammation. Tumor Necrosis Factor Superfamily Member 14 (TNFSF14/LIGHT) primarily acts on Lymphotoxin Beta Receptor (LTβR), promoting lung fibroblast division, upregulating IL-33 mRNA, and the expression of adhesion molecules intercellular cell adhesion molecule-1 (ICAM-1) and vascular cell adhesion molecule 1 (VCAM-1), inducing the transcription of CC or CXC chemokines in lung fibroblasts, amplifying inflammatory and fibrotic responses. Targeting the blockade of LIGHT activity may provide new therapeutic strategies for pulmonary fibrosis treatment (182). Furthermore, Zheng et al. (183) in an *in vitro* study, unveiled the association between IL-1 receptor-associated kinase (IRAK)-M and pulmonary fibrosis. The overexpression of IRAK-M exerts a beneficial influence on pulmonary fibrosis-related alterations: it notably impedes the proliferation and motility of lung fibroblasts, diminishes the expression of fibronectin, type I collagen, and α-SMA in Murine lung fibroblasts (MLg) cells, and boosts the production of metalloproteinases (MMP9) (183). Essentially, pharmaceutical agents that activate IRAK-M could emerge as a promising avenue for future investigations.

As previously discussed, the ongoing progress in medical research is deepening our comprehension of the pathophysiology of pulmonary fibrosis. Research on associated targets and pathways is progressively broadening, offering valuable insights for the future advancement of clinical practice.

### 5.5 Other respiratory diseases

In models of acute lung injury (ALI) and ARDS, elevated serum IL-33 levels contribute significantly to the pathogenesis, primarily through the IL-33/STAT3/MMP2/9 pathway (184). Studies have demonstrated that neutralizing IL-33 with specific antibodies can reduce the levels of MMP2 and MMP9, thereby mitigating the severity of lung injury associated with ARDS (184). Additionally, excessive cellular autophagy has been implicated in increased mortality due to ARDS-induced lung injury (185). Liang et al. (185) investigated the role of autophagy modulation in ARDS using rapamycin (RAPA), an autophagy promoter, and 3-methyladenine (3-MA), an autophagy inhibitor. Their findings revealed that 3-MA inhibits NF-κB-mediated IL-33 expression, leading to a reduction in the uncontrolled inflammatory response observed in ARDS.

HMGB1 is an evolutionarily conserved non-histone nuclear protein expressed in nearly all cells. In allergic airway diseases, HMGB1 promotes the release of IL-33 through the HMGB1/RAGE/IL-33 axis. Glycyrrhizic acid has been shown to inhibit HMGB1-mediated IL-33 release and attenuate lung injury (186). Genetic engineering approaches also hold promise for ARDS treatment. The ST2/T1 gene in human mesenchymal stem cells (hASC) can be selectively spliced using two different promoters to produce two major products: ST2L and sST2. ST2L is a functional component of IL-33 activity induction, while sST2 acts as a decoy receptor for IL-33, inhibiting TLR-4 expression and pro-inflammatory cytokine production by macrophages. Treatment with hASC-sST2 blocks IL-33 binding to ST2, resulting in a protective anti-inflammatory and immunomodulatory effect that reduces pro-inflammatory cellular infiltration and preserves pulmonary vascular barrier integrity (187). This approach offers a potentially effective option for ARDS treatment by inhibiting IL-33 expression and mitigating the inflammatory response.

## 6 Prospects and the future

In summary, IL-33, a cytokine of the interleukin family, has emerged as a critical player in respiratory type 2 inflammation and is pivotal in the development of asthma, allergic rhinitis, and various other respiratory diseases. This review has illuminated the pathways through which IL-33 contributes to respiratory disease development and has explored the mechanisms and feasibility of drugs targeting IL-33.IL-33 activates the ST2 receptor, initiating a cascade of inflammatory responses that drive the progression and aggravation of respiratory diseases. Therapeutic strategies targeting IL-33 involve blocking IL-33 binding to its receptor, suppressing IL-33 expression, and interrupting IL-33 signaling pathways. Certain drugs, such as monoclonal antibodies and small molecule inhibitors, have demonstrated promising therapeutic effects against IL-33. Looking ahead, further research into the role of IL-33 in respiratory diseases and the clinical application of IL-33-targeted therapies will be crucial. By deepening our understanding of IL-33 regulatory mechanisms, we can develop more effective and personalized treatment approaches, ultimately providing better therapeutic options and improving quality of life for patients with respiratory diseases.

We should recognize that there are currently many shortcomings in targeting the mechanism of action between IL-33 and type 2 inflammation-associated respiratory diseases. For example, the pathways of action have not yet been fully unraveled, as well as insufficient output in translational medicine research.

As an emerging research focus, understanding the role of IL-33 is crucial for gaining deeper insights into respiratory type 2 inflammation-related diseases. However, several challenges remain that require further investigation. Firstly, the mechanism of type 2 inflammation is intricate, and IL-33 represents just one component of this complex process. Exploring interactions with other inflammatory factors and assessing the comparative value of targeting these factors alongside IL-33 warrants future exploration. Secondly, identifying additional pathways influencing IL-33 and disease development requires comprehensive study and validation. To advance the translation of basic research findings, future studies can focus on several key aspects:

Biological Models and Biomarkers: Develop biological models to predict disease prognosis and progression by measuring IL-33 levels in serum or alveolar lavage fluid. Evaluate IL-33 as a potential biomarker for disease diagnosis and severity grading to enhance clinical guidance.

Clinical Trials: Conduct extensive clinical trials on drugs known to affect IL-33 production, release, and downstream pathways. Assess safety, human tolerance, drug distribution, and metabolism to inform treatment protocols and guidelines.

Precision Medicine: Employ personalized medicine approaches by analyzing IL-33 data from individual patients to identify suitable anti-IL-33 drugs, facilitating precision medicine initiatives.

Exploration of IL-33 in Other Diseases: Expand research on IL-33 involvement in type 2-related respiratory diseases like ARDS and pulmonary fibrosis. Investigate IL-33 production, pathways, drug efficacy, safety, and overall patient benefits to improve survival and quality of life.

In conclusion, IL-33 is intricately linked to various respiratory type 2 inflammatory diseases, and further investigation into IL-33 and related pathways holds promise for identifying novel strategies to prevent and manage type 2 inflammation.

Future studies should prioritize investigating undiscovered pathways of IL-33 production and action, assessing drug efficacy and safety, and exploring patient benefits to advance our understanding and clinical management of respiratory diseases associated with type 2 inflammation **Table 1**.

**Table 1:** Relevant drugs and their action mechanisms and effects.

Disease	Reference	Medicine	Target /Pathway	Main model/Experiment	Mechanisms and clinical role
Asthma	Zhu S, et al. (116)	Vitamin B6	PLP/MDM2/IL-33 axis	Papain-induced mouse model Human alveolar basal epithelial cell line A549 HEK293FT cell HDM-induced mouse model	Decreases IL-33 stability, promotes IL-33 degradation, reduces eosinophils and ILC2s, and attenuates type 2 inflammation
	Zhu G, et al. (117)	/	SPRR3/PI3K/AKT/NFkB	HDM-induced mouse model	Inhibits IL-33 expression, reduces ILC2s recruitment, and attenuates type 2 inflammation
	Shinoda K, et al. (118)	Ascr#7	Th 2 cells and ILC2 cells	OVA-induced mouse model HDM-induced mouse model	Reduces IL-33 expression and inhibits proliferation of ST2+ Th2 cells and ILC2 cells
	Galvão JGFM, et al. (120)	Wowbaine	Na+-K+-ATPase	OVA-induced mouse model	Inhibits IL-33 production and reduces inflammatory cell migration
	Won J, et al. (121)	IFN- λs	Th 2 cells	OVA-induced mouse model	Accelerates reduction of IL-33 levels
	Huang X, et al. (122)	Louki Zupa	IL-33/ST 2-NFkB/GSK3b/mTOR	OVA-induced mouse model	Inhibits IL-33, ST 2 expression and down-regulates inflammatory response
	Wuniqiemu T, et al. (123)	Iris flavin A	Th 2 inflammatory factors IL-4, IL-5, IL-33, leukocytes, epithelial transcription factor FOXA3 and mucin MUC5AC	OVA-induced mouse model	Reduces IL-33 expression, reduces infiltration of inflammatory cells and release of inflammatory factors
	Verheijden KA, et al. (67)	GOS	IL-33 mRNA、ST 2 mRNA	HDM-induced mouse model	Reduces IL-33, ST 2 mRNA expression
	Huang Y, et al. (124)	Thrombin inhibitors	IL-33 precursor	ALT-induced mouse model Papain-induced mouse model HDM-induced mouse model OVA-induced mouse model	Inhibits IL-33 precursor activation reduces eosinophils and ILC2s and attenuates type 2 inflammation
	Kimata M, et al. (126) Kempuraj D, et al. (127)	Mast cell inhibitors	Ca2+/PKC	Human cultured mast cells (HCMCs)	Inhibits IL-33 production
	Boberg E, et al. (129)	Rapamycin	mTORC1signaling pathway	IL-33 induced mouse model	Inhibits IL-5-induced bone marrow ILC2s
	Xiao Q, et al. (130)	Adenosine	Adenosine/A2A-cAMP and NFκB	Papain-induced mouse model	Inhibits ILC2s activation
	Toki S, et al. (131)	Glucagon-like peptide- 1 receptor agonist	GLP-1RA signaling pathway	ALT - EXT-induced polygenic obese mouse model (TALLYHO mice) and corresponding lean gene control mice (SWR mice)	Reduces acute release of IL-33 and attenuates activation of ILC2s
	Cavagnero KJ, et al. (132)	Cyclic-di-GMP	Interferon gene-stimulating factor (STING) and type 1 IFN signaling pathway	ALT-induced mouse model	Modulates ILCs response
	Yang Q,et al. (132)	Osthole	IL-33/ST 2 signaling pathway	OVA-induced mouse model	Inhibits IL-33/ST 2 signaling
	Liu G, et al. (188)	/	Fibulin-1	HDM-induced mouse model	Reduces IL-33 level
	Kelsen SG, et al.	Astegolimab	ST 2	Multicenter, randomized, double-blind, placebo-controlled, phase 2b study	Selectively inhibits ST 2 , reduces acute asthma exacerbations in a broad population of patients with severe, uncontrolled asthma
	Wechsler ME, et al.	Itepekimab	IL-33	Multicenter, randomized, double-blind, placebo-controlled, parallel, phase 2, proof-of-concept trial	Selectively inhibits IL-33, reduces the rate of uncontrolled moderate-to-severe asthma, and improves pre-bronchodilator FEV1
	England E, et al. (149)	Tozorakimab	RAGE / EGFR	ALT-induced mouse model	Blocks IL-33(red) and IL-33(ox) signaling, with good safety and tolerability
Allergic rhinitis	Zhang JJ, et al. (144) Kitano M,et al. (143)	Xiaoqinglongtang	MYD88/IRAK4/NF-κB P65	OVA-induced mouse model TDI-induced mouse model	Reduces IL-33 level
						Shaha A, et al. (146)	Betuletol	PKCδ/ERK and/or IL-33/ ST 2/ERK	PMA-induces swiss 3T3 cell	Down-regulates IL-33 mRNA expression
	Islam R, et al. (145)	Wild grapes	PKCδ	TDI-induced mouse model PMA-induces swiss 3T3 cell	Down-regulates IL-33 mRNA expression
	Long S, et al. (148)	/	miR-181a-5p/IL-33/ST2-p38 MAPK	OVA-induced RPMI-2650 cell	Targeted downregulates IL-33 expression
	Jin J, et al. (149)	Chaenomeles sinensis extract	IL-33/ST 2 signaling pathway	OVA-induced mouse model	Reduces IL-33 levels and inhibits IL-33/ST 2/Th 2 signaling
	Jin J, et al. (150)	F.japonica root extract	IL-33/TSLP/NF-κB	OVA-induced mouse model	Dose-dependent reduces IL-33 level and ST 2 mRNA expression
	Chronic obstructive pulmonary disease	Lee K,et al. (159)	Picroside II	SAA-TLR2 - NF-κB SAA-TLR2 - MAPKp38 - ERK1 / 2	SAA-induced human airway epithelial cell	Inhibits IL-33 production
Katz-Kiriakos E, et al. (160)		GW6849, inhibitor of nSMase2	nSMase 2	ALT-induced human airway epithelial cell	Inhibits IL-33 secretion
Rabe KF, et al. (161)		Itepekimab	IL-33	Multi-country, randomized, double-blind, placebo-controlled, parallel, phase 2a trial	Selectively inhibits IL-33, reduces exacerbation rates and improves lung function in COPD patients who quit smoking
Yousuf AJ, et al. (162)		Astegolimab	ST 2	Single-center, randomized, double-blind, placebo-controlled, phase 2a clinical trial	Selectively inhibits ST 2 to improve patients' respiratory function and quality of life
England E, et al. (137) Reid F, et al. (163)		Tozorakimab	RAGE / EGFR	ALT-induced mouse model Non-clinical, phase I, randomized, double-blind, placebo-controlled study	Neutralizes IL-33, forms IL-β 33(red)/tozorakimab complex, reduces IL-33/ST2 formation, and has a favorable safety and tolerability profile
Acute respiratory distress syndrome	Lei M, et al. (185)	3-methyladenine	NF-κB	LPS-induced mouse model	Reduces IL-33 level
	Fu J, et al. (186)	Glycyrrhizin	HMGB1	LPS-induced mouse model	Reduces IL-33 level
	Martínez-González I, et al. (187)	Human adipose tissue–derived mesenclymal stem cells overexpressing soluble IL-1 receptor–like–1	IL-33	LPS-induced mouse model	Blocks IL-33 binding to T1/ST2
Pulmonary fibrosis	Yi XM, et al. (87)	/	USP 38 and TRAF 6	Defective Usp38 in Embryonic Fibroblast NIH 3T3 Cells Usp38 Deficient Mouse Model Bleomycin-Induced Mouse Model	Modulating the levels and activity of IL-33R regulates signal transduction, inflammation, and fibrosis.
	Zhang JJ, et al. (85)	/	NRP 1	Id2cre-ert2Nrp1f/f Mouse Model Bleomycin-Induced Mouse Model R5/+ Nrp1f/f Mouse Model	Deficiency in NRP1 downregulates ST2, weakens ILC2 function, and alleviates pulmonary fibrosis.
	Nie YJ, et al. (89)	/	NFATc 3	NFATc3-deficient mice Bleomycin-induced mouse model	Deficiency in NFATc3 significantly reduces the levels of fibrotic markers CCL2 and CXCL2 induced by BLM, attenuating BLM-induced pulmonary fibrosis and inflammatory response.
	Gao YX, et al. (83)	Pyrrolidine dithiocarbamate (PTDC)	NF-κB signaling pathway/eIF3a	Bleomycin-induced mouse model.	Inhibit the expression of eIF3a induced by NF-κB pathway activation, thereby alleviating pulmonary fibrosis.
	Nie YJ, et al. (189)	Elamipretide	NRF 2/NLRP 3	Bleomycin-induced mouse model for research purposes.	Inhibit the activation of the NLRP3 inflammasome mediated by NRF2 in macrophages.
	Xiong Y, et al. (178)	Dehydrocostus lactone	JNK/p38 MAPK/NF-κB	A bleomycin-induced mouse model.	Downregulate the NF-κB signaling pathway mediated by JNK/p38 MAPK, reduce the expression of inflammatory cytokines induced by BLM, inhibit macrophage activation, ameliorate lung inflammation, and alleviate pulmonary fibrosis.
	da Silva Antunes R, et al. (182)	/	TNFSF14	Primary human lung fibroblasts (HLF).	LIGHT promotes the proliferation of lung fibroblasts, upregulates the expression of cytokines, amplifying inflammatory and pulmonary fibrotic responses.
	Zheng ZD, et al. (183)	/	IRAK-M	Mouse lung fibroblasts (MLFs).	Inhibit the proliferation and motility of lung fibroblasts, reducing the expression of fibronectin, type I collagen, and α-SMA in MLg cells.
